# Perspectives on pregnant women’s educational needs to prevent TB complications during pregnancy and the neonatal period. A qualitative study

**DOI:** 10.1186/s12889-023-16770-w

**Published:** 2023-10-13

**Authors:** LB Khoza, SA Mulondo, RT Lebese

**Affiliations:** 1 Department of Health Studies, University of South Africa, Pretoria, South Africa; 2 Faculty of Health Sciences, University of Venda, Thohoyandou, South Africa

**Keywords:** Tuberculosis, Pregnant women, Maternal morbidity, HIV co-infection

## Abstract

**Background:**

Tuberculosis (TB) during pregnancy could confer a high risk for maternal and infant morbidity. Literature indicates that the global burden of active TB disease among pregnant women is not well researched. Statistics for South Africa from WHO give an estimated incidence of 360, 000 cases of TB in 2019; 14,000 people became ill with multidrug-resistant TB in 2019, with a rate of 615 per 100,000 population, implying that the cohorts included pregnant women with and without a diagnosis of TB infection. Therefore, the study aims to increase the understanding of the educational needs required to prevent TB complications during pregnancy and the neonatal period in women diagnosed with TB infection.

**Methods:**

The study used cross-sectional qualitative and descriptive designs to collect data in the clinical setting of the primary health care services of Limpopo Province, South Africa. The population comprised pregnant women diagnosed with TB infection. A non-probability purposive sampling technique was used to sample 2 health centers and 5 clinics in each of the three sampled districts. The targeted sample size was 63 and it was achieved even though data saturation was observed. Individual interviews were conducted, audiotaped, and transcribed. Guided by the study questions, a thematic content analysis of the findings was used. Ethical considerations were also observed.

**Results:**

Despite that pregnant women have general knowledge about TB disease, the knowledge and awareness regarding the prevention of TB complications in pregnancy and the neonatal period, information on TB/HIV and COVID-19 co-infections, and participants’ knowledge about other non-infectious diseases that may affect the mother with TB infection and foetus showed a deficit.

**Conclusion:**

Pregnant women with TB disease need to be educated on the negative effects of non-adherence to TB treatment during pregnancy and the neonatal period. There is a need to educate pregnant women about the variant signs and symptoms of TB, HIV and COVID-19 infections since there is a misconception that the three diseases are similar. It is important that pregnant mothers diagnosed with TB should start treatment as soon as possible.

## Background

Tuberculosis is an infectious disease that is a major cause of ill health and one of the leading causes of death globally. The World Health Organisation (WHO) estimated that more than 2 million people died in 2018 [1, 2]. Africa alone is estimated to have a TB incidence of 2.5 million out of 9.9 million globally. The updated WHO report reveals that Europe has the lowest incidence of 231, 000 followed by America and the highest TB incidence of 4,37 million is in South-East Asia [3]. In 2020, South Africa was one of the 30 high-burden TB countries that collectively contributed above 86% of the estimated incident TB cases worldwide, and eight of these countries accounted for two-thirds of the global total namely India (26%), China (8,5%), Indonesia (8.4%), the Philippines (6.0%), Pakistan (5.8%), Nigeria (4.6%), Bangladesh (3.6%) and South Africa (3.3%) [4, 5]. Among the 30 burdened countries South Africa is among the 14 countries with the highest burden of coinfection HIV/TB and multi-drug resistant TB (MDR-TB) [6].

In highly burdened countries active TB disease during pregnancy remains associated with a substantially elevated risk for poor maternal and foetal outcomes, including a threefold increase in morbidity, antenatal admission, anemia, and caesarean births, a ninefold increase in miscarriage, a twofold increase in preterm birth and low birth weight and a sixfold increase in perinatal death] [7, 8]. Poswa, et al., [9] indicate that TB infection during pregnancy has been reported to be associated with maternal and foetal adverse effects and has not been critically elucidated.

Literature indicates that the global burden of active TB disease among pregnant women is not well-researched [10, 11]. The adverse maternal and neonatal outcomes are increased with inadequate treatment, advanced disease attributed to late presentation, and diagnosis of TB in pregnancy compared with earlier diagnosis [7, 12]. Miele, et al., [10] indicate that TB is most common during a woman’s reproductive years and is a major cause of maternal-child mortality. The authors explored the global burden of TB in women of reproductive age and found that TB kills approximately 500,000 women annually, a disproportionate number of them during their reproductive years [10].

The risk of untreated active TB disease in pregnant women and foetuses is greater than the risk of taking treatment [10]. Most common problems of congenital TB may be transmitted from the mother with an active TB disease through the placenta to the foetus. Although research shows that TB medications cross the placenta, these drugs do not appear to have harmful effects on the newborn [10]. Although congenital TB is a rare complication in utero TB infection, it has a high mortality rate as reported by Gould and Aronoff [12] it may present in the early neonatal period with sepsis or the first three months of life with bronchopneumonia and hepatosplenomegaly [12, 13]. The major problem concerning TB diagnosis for pregnant women is the delay in diagnosis and late presentation at antenatal care. TB increases mortality during pregnancy and postpartum, especially in HIV-positive women [13]. Women with pulmonary or extra-pulmonary TB also have an increased risk of complications including hospitalisation and miscarriages [14].

In India, TB kills more mothers of reproductive age than any other cause of maternal mortality. More than 80% of the mothers with TB are in the reproductive age group of 15 to 54. The effects of TB on pregnant women depend on the organs of the body affected, the extent of the disease, the stage of pregnancy, the nutritional status of the mother, immune status and the existence or non-existence of HIV infection, as well as the availability of facilities for early diagnosis and treatment of TB [14]. Although Limpopo and Gauteng provinces in South Africa are classified under a low-burden stratum, in Limpopo, TB in pregnant mothers could be aggravated by the low levels of education, HIV/AIDS infection, increasing poverty, and resistance to anti-TB drugs [4, 15].

In South Africa, the National Department of Health introduced Basic antenatal care (BANC) as the minimum ANC service provided to every pregnant mother. This was introduced to alleviate the burden of a consistently high maternal and perinatal mortality rate. BANC is a minimum level of ANC that all pregnant women should receive to improve maternal and neonatal outcomes [16]. The BANC services engage expected mothers, other family members, friends and midwives in the healthcare decision [16, 17]. BANC guidelines stipulate five visits with the first visit occurring within the first 12 weeks of pregnancy. HIV counselling and testing are done during the first visit, followed by TB screening. ANC visits are then increased for women at high risk of HIV/TB infection. All pregnant women are encouraged to initiate ANC services early within the first three months of pregnancy for early detection, diagnosis and treatment of conditions such as TB [18, 19]. Despite the implementation of BANC services in South Africa, mothers, mostly in rural villages of the provinces start ANC services late during or after the second three months of pregnancy [19, 20]. In Durban, South Africa, infants born to mothers diagnosed with TB were at the highest risk of TB infection because their mothers presented late at ANC or had not received appropriate prenatal care [21].

The WHO provides statistics for South Africa that there was an estimated incidence of 360,00 cases of TB in 2019, 14,000 people became ill with MDR- TB the same year at the rate of 615 per 100,000 population. Although the report shows that Limpopo province ranked fifth of the nine provinces with an incidence rate of 301 per population (2015), there appeared to be little publicity available information regarding the incidence rate for pregnant women with TB in Limpopo. The SA Short Report (2018) further indicates that there is a high TB burden including many people with an undetected disease in the community [4]. The report shows that the highest percentage of symptomatic participants (79%) who did not seek care was between the ages of 25 and 34, implying that the cohorts included pregnant women diagnosed and undiagnosed with TB infection [4, 22]. The main study conducted by the primary researcher in Limpopo province concluded that pregnant women with TB infection lacked knowledge regarding late presentation to ANC service, TB complications, and associated TB symptoms with HIV [11]. Therefore, the current study aims to increase the understanding of pregnant women’s knowledge regarding TB disease and its complications during pregnancy and the neonatal period. The following questions were sought.

- What are the educational needs to prevent TB complications in pregnancy and neonatal period?
- To what extent pregnant women are informed about TB/HIV and COVID-19 co-infections?
- What knowledge pregnant women have about other non-infectious diseases that may affect the mother with TB infection and the foetus?

## Methods

### The setting of the study

The study was conducted in the clinical setting of the primary health care services of Limpopo Province, South Africa. Limpopo Province is divided into five districts and subdivided into 26 local municipalities. The five districts are Capricorn, Mopani, Sekhukhune, Vhembe and Waterberg district. The province’s total population is estimated to be 5,9 million [23]. The population of Limpopo consists of several ethnic groups distinguished by culture, language and race. The most spoken languages are Sepedi, Xitsonga and Tshivenda. Because of its proximity to the South African Development Community countries, namely, Zimbabwe, Botswana and Mozambique, many immigrants illegally cross the borders to Limpopo. The influx of foreigners not registered by Home Affairs in South Africa could have contributed to the high burden of pregnant women with TB disease, particularly in the three districts where the current study was conducted. The estimated black population is 96.7%, the white (2.6%), coloured (0.3%) and the Indian/Asian population (0.3%). The current study provides the meaning of coloured population as persons of mixed descent, European (white) and African (black) as officially defined by the South African government from 1950 to 1991. Out of the five districts, three were identified as crisis districts for TB namely, Mopani, Sekhukhune and Vhembe [24].

### Design, population, sampling and sample

The study used a qualitative descriptive design, to give a holistic meaning and understanding to the phenomenon as it exists in the real world. All decisions made in planning the study, such as identifying the topic to be studied, target population, research methods and for what purpose, were included. This guided the researchers in planning and implementing the study in a way that was most likely to achieve the intended objectives [25].

Population refers to the set of individual units, organisation units, case records, or other sampling units with which the research problem is concerned, and the research questions seek to find out about the topic under study [25]. The population should conform to a set of specifications having some common characteristics that would provide relevant data about the study [26]. Polit and Beck [27], and Burns and Grove [25] describe the target population refers to the total group of subjects about whom the researcher is interested and about whom research findings can reasonably be generalised. The accessible population is a portion of the target population that is accessible to the researcher as a pool of subjects. The accessible population in this study comprised pregnant women diagnosed with TB infection.

Sampling is the process of selecting a few participants from the entire population to gather data or to be observed from the population under observation [28, 29]. We used a non-probability sampling approach to select the sample of primary health care centers and clinics, and pregnant mothers diagnosed with TB. The Department of Health administers 44 hospitals, 27 health Centers and 410 clinics [24]. Based on the highest number of pregnant mothers with TB taking medication in the PHC facilities, two health centers and five clinics rendering maternal health care services were purposively sampled in each of the three sampled districts. The overall sample comprised 6 health centers and 15 clinics that were visited for data collection.

A non-probability convenient sample of pregnant women diagnosed with TB was selected as participants. The TB coordinator was instrumental in informing us of the days and times likely to have a pick number of pregnant mothers coming to ANC services. The participants were available at the time in question because they were present for TB treatment refills, consultation about other health-related issues, and attending ANC centers; some were admitted to maternity units for observation. Participants were interviewed as they arrived for such services. This study aimed at selecting 3 participants, diagnosed with TB and on TB treatment from each PHC facility, implying that 21 respondents were selected per district. The total number of participants would be 63 or guided by the point of data saturation during interviews.

### Data collection

Guided by the three questions formulated in this study, an individual semi-structured interview tool was developed. Due to the sensitivity inherent and associated with HIV and disease stigma in the community, focus group discussion was not a preferred approach to collect data. The nurse manager of the facility arranged a private room for interviews to be conducted. The quality of the responses from participants was enriched by probing questions that were elicited by the individual participants. Therefore, variations were expected from individual experiences and perceptions regarding the phenomenon. Data collection was completed over four months that is, between March to June 2022.

The three research assistants who were professional midwives acquittance with the three common languages in the Mopani, Sekhukhune and Vhembe districts of Limpopo province, were appointed. All three assistants had master’s degrees and studied toward a doctoral degree. Engaging them in this study served as an empowerment strategy to benefit their research endeavours. Research assistants were briefed on the research project, the purpose, questions of the study, the instrument to be used and trained in the use of the voice recorder and probing tactics. In addition, observing the principles of autonomy, respect, confidentiality, beneficence, and maleficence was emphasised. The voice recorder for recording discussions before the commencement of the interview was checked to ensure that it was in good condition and those extra batteries were available. Notebooks and pens were also available for research assistants. Copies of the informed consent form and interview guide were prepared. A note to thank participants for their contribution to this study was also prepared.

### Data analysis

Three different language experts made the translation of the participants’ transcripts from the traditional languages to English. The English transcripts were proofread and positioned thematically. The study followed thematic coding to analyse the semi-structured interview data. The study questions constituted the themes of generating initial codes, 2) searching for sub-themes, 3) reviewing themes and sub-themes, 4) defining and naming the themes, and 5) producing a report [27].

### Ethical consideration

The Research Ethical Committee of the University of Venda approved the study (Ref: SHS/19/PDC/34/0511). Additionally, the Limpopo Department of Health and the Mopani, Sekhukhune, and Vhembe Districts Municipality’s health department granted permission to conduct the study. Participants were fully informed about all aspects of the study by reading and translating information into their language in the consent letter before they consented to participate. They were assured that the information shared would not be linked to any of them as pseudonyms would be used if needed. They were informed that they could terminate their participation at any stage of the interview if they became uncomfortable. Participants were further informed that confidentiality and anonymity would be maintained and that their recorded voice equipment and written notes would be kept safe in a locked steel cabinet and only the primary researchers would have access if where needed.

### Trustworthiness

Trustworthiness is when the study accurately represents how the researcher convinces the readers that the findings are worth taking note of. Trustworthiness can also be considered as the degree of confidence that qualitative researchers have in their data, and it is evaluated using the criteria of credibility; transferability; dependability; and conformability [27]. In this study, credibility was ensured through techniques such as prolonged engagement, quality probing questions, clarification of researcher bias, peer debriefing, and in-member checks [30]. Pretesting of the developed interview guide was conducted with experts in the health discipline to determine whether the proposed study would be feasible and to determine the clarity of the interview questions. Transferability refers to the degree or extent to which the findings from the data can be applied to other contexts and settings [30]. It is the ability to generalise the findings to a larger population. In the study, context generalisability applied to the entire Limpopo province of South Africa. Three of the five districts in Limpopo province comprised the sample of the current study based on the highest number of TB infections among women of childbearing age. Dependability refers to “the stability of findings over time” [30–32]. Dependability involves participants evaluating the findings and the interpretation and recommendations of the study to make sure that they are all supported by the data received from the informants of the study [31, 33]. Dependability was achieved through auditing of research processes. Lastly, conformity was established through the involvement of an expert in qualitative research who reviewed the thematic coding of the data guided by the study questions, the findings, and interpretations to test conformability.

## Results

The study findings are presented under (1) biographical characteristics of the participants, (2) participants’ perspectives on their educational needs to prevent TB complications in pregnancy and the neonatal period, (3) participants’ perspectives on their required information on TB/HIV and COVID-19 co-infections and (4) participants’ perspectives on knowledge about other non-infectious diseases that may affect the mother with TB infection and unborn baby.

### Biographical characteristics of the participants

The age range of the participants was between 18 and 43 years, with the highest (52) between 21 and 40 years. Out of 63 participants interviewed, 40 were married and 23 were not married. Only 13 of the participants were primigravida, and 9 were having multiple pregnancies ranging between 5 and 7. The participants who were within the BANC guideline (first three months) for presentation at ANC service were only 24, whereas most women attended ANC services in their second and third trimester of pregnancy, and that would be considered late presentation, delayed in starting TB treatment and could have been in the progressive stage of TB infection. Most importantly most participants (27) were illiterates, 30 of the participants did not attend secondary education, whilst only 6 of the participants were at post-secondary higher education institutions during the period of data collection.

### Participants’ perspectives on their educational needs to prevent TB complications in pregnancy and neonatal period

The study findings provide an understanding that the participants have basic knowledge about the importance of attending ANC services, screening for diseases such as high blood pressure, blood sugar, TB and HIV infection, and the importance of diet, exercise, and a healthy lifestyle by avoiding alcohol and smoking and taking of TB and HIV medication during pregnancy to avoid giving birth to an infected baby. For instance, one participant mentioned that *‘early presentation to ANC services saved me and my second born baby’ life. I started coughing and sweating at night and I was ‘so’ thin. My mother took me to this clinic and nurses screened me for HIV first, it was positive, they took my sputum and came back also positive. They started me with many tablets which made me vomit sometimes but taking five pills daily did not bother me at all because I was told that if I don t, I and my baby would die ****(27 years old).*** The participant was asked except for dying what information she knows about the complications that might arise in pregnancy and the newborn baby. The participant emphasised that if a mother takes treatment regularly there would not be any complications arising for the baby because she delivered her first baby while on HIV medications and the baby was born free of the two diseases.

Most participants could not articulate adequately how TB can affect the unborn baby, the importance of testing for TB, COVID-19 and HIV during ANC services, complications associated with TB during pregnancy and the antenatal period, and why is it important to an early presentation at antenatal care services, information about TB as a disease, on how to prevent and manage maternal TB. A mother shared her experience of late presentation at the ANC services and how her health deteriorated drastically and mentioned that ‘*I visited the nearby this clinic when I realise that traditional means of relieving cough and difficulty in breathing could not help me. I started coughing early in pregnancy but because everyone in the village was using steam to cure coronavirus, I also thought it was corona symptoms it would pass. My symptoms were getting worse over time, and I did not think of TB infection until it was discovered in my third trimester when I started the ANC services’ ****(40 years old, grand-multiparous).*** The participant was asked about healthcare workers’ lessons about TB infection in pregnancy. She further mentioned that *‘I was told that it is possible that my baby will be born with TB infection and will also have to be given TB medication when born’*. The participants’ expressions could tell that women infected with TB are not adequately taught about the TB complications that might affect the mother and the baby. Another participant just said, ‘*Eish! I am not sure about the complications! To be honest with you I do not know’ ****(29 years old).***

Participants expressed the need for education on TB as a disease and how it affects the mother and the unborn baby. Participants felt that giving the health talk by nurses combined with other patients who come for general consultation deprives them of learning more about TB during pregnancy. Most participants did not know about MDR-TB as a complication of TB and that TB may present in the early neonatal period with sepsis or the first three months of life with bronchopneumonia and failure to thrive and that early TB screening and diagnosis is fundamental.

A Participant said, ‘*Educational needs regarding TB disease in pregnancy are important. We need to be educated about the TB complications that might arise affecting me and my baby. Nurses are too superficial to educate us because we are mixed with all patients coming for consultations. It seems nurses do health education as part of their routine, which is unfair to us. Knowing what will happen to me or the baby, why should I adhere to TB treatment, and how it will affect my baby, is important to know but nurses are always in a rush’ (****21 years old,).*** It was pleasing to find that the participant indicated that since she was diagnosed with TB/HIV co-infection, she became inquisitive to know more about the relatedness of the two diseases by seeking information in books and brochures and even listening to the media. The participant further indicated that adherence to both TB/HIV for the prevention of mother-to-child transmission was her priority.

Another participant said: *“Women need to come to the health facility and be taught about complications of TB in pregnancy even if they are not pregnant. This information is very important to know.”* paused and further said: *“‘In fact, all other women of childbearing ages, in schools to educate our girl children, and the community at large. We have home-based health workers in the community who can educate us about this deadly TB disease’ (****32 years old). ****C*onsequently, some participants did not see the need to take TB medication regularly for a prolonged period including after delivery because it made them vomit and experience dizziness. One participant shared her experience of seeking traditional medicine to stop vomiting during pregnancy and said, ‘*every pregnant woman has vomiting, like me, despite using herbal medicine given by the traditional practitioner, vomiting continues until I give birth. I will start to take TB tablets when I have delivered the baby as there is no risk of transferring medicine to the baby and causing more harm’**** (39 years old, multiparous).*** The finding could conclude that the participant lacked knowledge that the positive effects of TB treatment on the unborn baby and her, surpass the TB complications that both could experience.

### Participants’ perspectives on their required information on TB/HIV and COVID-19 co-infections

Participants demonstrated more knowledge about TB/HIV co-infection. They were able to describe the different modes of transmission, and symptoms and that HIV infection needs lifelong treatment whilst TB is curable. Participants expressed that pregnant women need to get information regarding testing for TB and HIV at ANC services. one participant said:

*“We need information on TB screening and testing at ANC services, and HIV testing because these two diseases are similar”.* Pause, *‘but the challenge is that nurses do not do screening when we come for booking. Especially these days of COVID! Nurses do not want to spend much time with you’.*

Participants further expressed concerns that TB and HIV infections have a stigma in the community, and if a person thinned out and started coughing, the community discriminated against her. The study findings indicated that there were still some myths among the participants. Older participants associated TB and HIV symptoms with ancestral calling, they would consult traditional ‘sangomas’ meaning healers to initiate training. One participant mentioned that ‘*ancestral spirit told me that going to the hospital for consultation because I was very sick would aggravate the condition and I and the baby would die.* Showing some regret signs on her face, she further said, ‘*I was brought to the hospital in a critical stage, no longer knowing who I am, legs were swollen and with difficulty in breathing. Later, the nurse told me that I was having TB/HIV infections together. Somebody came to teach me what it means to have TB/HIV co-infection and said that I will be given treatment to save the unborn baby, I just prayed to save my baby’**** (37 years old).*** Another participant said, ‘*You know, we are from different families, some do get support because they understand the disease, others do not get support because they believe that TB and AIDS are associated with witchcraft, so you must be taken to the traditional healers for treatment”.* Pause, *“so is a problem because using traditional herbs makes one stop taking the TB and HIV drugs’.*

Participants explained different perceptions regarding the myths and during probing, most participants concluded that women should be educated on the consequences of ignoring symptoms of both TB and HIV infections, risk factors, and health-seeking behaviours immediately in the first trimester of pregnancy.

### Participants’ perspectives on knowledge about other non-infectious diseases that may affect the mother with TB infection and unborn baby

The finding revealed that all the participants are somewhat knowledgeable about the most common conditions during pregnancy such as high blood pressure, swollen legs, and diabetes including minor disorders such as nausea and vomiting, urine frequency, and dizziness. Some participants were aware of the adverse effects of high blood pressure and diabetes indicating that the mother and baby could die, or the baby born with some deformities if the conditions were not managed during early pregnancy. Participant aged 29 years said, *‘I know that there is high blood pressure, HIV/AIDS, Coronavirus, vomiting or maybe many diseases I do not know’* She laughed and continued to say, *‘Ah! These are the only diseases I know of, but I don’t have any of them except TB, I presented early to*.

*attend ANC services, and I am on TB treatment, hence I feel good’.* Another participant said, *‘When coming for ANC booking, on your first day, nurses ask you about any person in the family suffering from Epilepsy, diabetes mellitus, or twin pregnancy. Another common condition that I know troubling pregnant women is high blood pressure’*.

Most participants indicated that coping with non-infectious diseases is better, than their experience of coping with the COVID-19 pandemic. Participants expressed that they lacked knowledge about the disease and how it affects unborn babies. Participants shared their fears about COVID-19, and they expressed that it was killing many people including pregnant women who were living with them in the communities. A participant mentioned *‘Yaa, presently we are having coronavirus, it is also a problem even to pregnant women and I don’t know if those who died with coronavirus were also using traditional steaming. Anaemia, HIV and AIDS, and malaria are also troublesome during pregnancy’**** (23 years old).*** However, there were unfounded morbidity statistics related to COVID-19 deaths among pregnant women in Limpopo province.

## Discussion

Since this study was conducted in rural and poor socio-economic environments of Limpopo province, the finding revealed a high illiterate rate among the participants and a poor understanding of TB complications during pregnancy and the neonatal period.

### Participants’ perspectives on their educational needs to prevent TB complications in pregnancy and neonatal period

The current study’s findings increased the understanding that the participants are well-oriented with the general knowledge of TB including that it affects the lungs, its signs and symptoms and that taking a long-term treatment is curable. However, participants lacked knowledge or had a poor understanding of ANC service benefits such as early diagnosis and commencement of TB treatment could prevent complications during pregnancy and the neonatal period, and associated TB symptoms with HIV and COVID-19 infections. The study further revealed that the participants who experienced cough, shortness of breath, lack of appetite and sweating at night did not go to the clinics instead they practiced traditional steaming to treat COVID-19 infection. The participants further raised a misconception about seeking medical attention by indicating that TB and HIV are caused by witchcraft and COVID-19 symptoms are treated by herbs, and most COVID-19-positive patients who have been hospitalised died. Most participants were from a poverty-stricken environment coupled with low educational standards. This finding could have contributed to their level of understanding of the risks associated with TB disease. A similar finding was revealed in South Asian countries, that there is a close link between TB, poverty, and illiteracy. It is very important to understand the potential effects of these factors on maternal and perinatal health, especially in low-socioeconomic countries [34].

Solarin and Black [35] confirmed that in South Africa, the average rate of ANC coverage is as high as 97% however, most pregnant women are seen once, either by Public or General Private Practitioners, which contradicts the four ANC visits, excluding the first visit, recommended by the WHO. Mulondo and Khoza [20] also found that rural women in Limpopo ignored symptomatic TB and did not see the need to attend ANC services earlier. Another study was conducted at Ravensmead, Cape Town in South Africa about the knowledge, and beliefs about TB and late presentation at ANC services. The findings reveal that pregnant women were aware that TB is a problem, knew that it affects the chest, knew that it is infectious and knew that local clinics provide treatment, however, ignored the need for early presentation at ANC services [36]. A similar general knowledge was addressed in the current study however, participants in both studies lacked knowledge on the adverse effect of TB on the mother and the unborn baby and that late presentation at the ANC services may lead to TB complications contributing to high maternal and neonatal morbidity was found.

Similar findings were reported from Ghana, Kenya and Malawi in sub-Saharan countries that mothers initiated ANC services after the second three months of pregnancy and did not achieve the recommended number of ANC visits [37]. In these countries, the ANC is considered a means of general checking of the foetus’ position as a result only motivated mothers attend ANC services earlier. Most pregnant women attend ANC to obtain ANC cards to avoid being reprimanded by health workers. The study conducted in India found that pregnant women over the age of 34, with or without comorbidities, were found to have a higher risk of developing pulmonary complications [38].

The current study found that most participants are entrenched in their cultural beliefs and practices related to pregnancy and TB as a disease that could be treated by traditional herbs and that a grand multipara woman could start ANC services anytime during the second or third trimester if she feels well. The study found that pregnant women presented late at the ANC services because TB symptoms were associated with witchcraft therefore, they would start by seeking traditional healing before coming to the clinic, and most importantly due to stigmatisation of the disease. Similarly, the findings of the study conducted in Cape Town, South Africa also revealed misconceptions about the causes and transmission of TB and the stigmatisation of the disease. The results further revealed that 46% of mothers indicated that neighbours did not wish to associate with mothers with TB for fear of infection. Most mothers (79%) presented late at ANC services owing to misconceptions about transmission. Poor relationships between pregnant women infected with TB and non-infected people may increase the stigma associated with TB, contributing to late presentation for ANC [36]. Another study in India also showed that more than half of the study population had inadequate knowledge of TB an.

“d close to half had a stigmatising attitude towards TB [34, 39].

Reports of the studies conducted in Nigeria indicate that some cultures perceive pregnancy as a normal-natural process of life. Therefore, pregnant women, immediate family members and community members underestimate the importance of ANC services owing to a lack of knowledge and awareness about PHC services including dangerous signs during pregnancy. Most pregnant women find themselves in a dilemma of not knowing how, when, and where to seek care if any complications arise during pregnancy [40]. It has been reported in the literature that anti-TB treatments may cause menstrual irregularities leading to some pregnant women not realising that they are pregnant [39]. In abdominal TB, amenorrhoea may mimic pregnancy [39, 41]. This can cause some pregnant women not to seek ANC services earlier as they might not be sure of their pregnancy [39]. In India, Zambia and Cameroon, late presentation for ANC services was also associated with side effects [41, 42].

### Participants’ perspectives on their required information on TB/HIV and COVID-19 co-infections

Several studies reported that TB and HIV are inextricably related and the negative impacts of each on the other have been widely documented [10, 35, 39]. HIV and TB infections during pregnancy are considered a ‘deadly combination’ by Jana et al. [34] and are independent risk factors for maternal morbidity in underdeveloped countries including Africa.

Despite those participants in the current study demonstrating basic knowledge of TB and HIV infections, the finding indicated a knowledge gap concerning risks associated with the late screening of TB and HIV in pregnancy may lead to serious complications including mother and child morbidity. The intersecting epidemics of HIV, COVID-19 and TB were regarded as major aggravating factors in the current study. Participants expressed concern that nurses are not doing a thorough screening during ANC visits because they fear contracting coronavirus. Most participants indicated that nurses would ask them if they had any complaints without touching them to feel the baby (meaning abdominal palpation) or ask if they were coming for a treatment refill. COVID-19 was a scare for all participants who required more knowledge and understanding of how the virus could affect the pregnancy and the unborn child. Although there is little published research work regarding how COVID-19 influenced pregnancy outcomes in South Africa, Kotlar et al., [43] conducted a systemic literature review study and found that there is evidence that pregnant women experiencing symptoms of COVID-19 are at higher risk of adverse outcomes than those who are not pregnant [43–45]. The authors [43] further indicate that pregnancy may constitute a particularly vulnerable period for COVID-19, however, the assumption requires further confirmation through research [43]. Similarly, the study conducted in India states that the risks to the foetus due to COVID-19 during pregnancy are not fully known, however, co-infection with TB could further complicate the situation [38]. The study conducted in the six provinces of South Africa found that the maternal mortality rate was high among women admitted with SARS-CoV-2 infection in low-income settings with TB as the only co-morbidity associated with admission [46]. However, the results could be generalised to other provinces of low socio-economic background such as Limpopo. In this study, the participants’ expectations of receiving more information related to the adverse effects of COVID-19 on the mother during pregnancy could be inadequately transferred, as more scientific research should be expanded.

In the current study, participants indicated that intense screening for TB was done on women with symptoms of the disease by referring them to the hospital for further management. It is evident that patent and asymptomatic patients are likely to be missed and TB progresses affecting both the mother and the unborn baby. The study further indicates that there is a need to demystify the myths entrenched in the participants’ culture and beliefs associated with TB and HIV infections. The stigmatisation of the said diseases could be regarded as a contributory factor for the late presentation at the ANC services. Counseling strategies should be developed to assist the affected pregnant women to build self-confidence and make them understand that with early diagnosis and treatment HIV and TB infections complications could be prevented. Munro, Lewin, Smith, Engel, Fretheim, et al. [47] indicate that there is some evidence that where there is TB/HIV co-infection, a diagnosis of TB may be seen as a diagnosis of HIV, and as a result, TB may be regarded incurable, or not treated [47]. Miele et al. [10] state that pregnant women living with HIV need to be screened for TB early in pregnancy and evaluated thoroughly to initiate TB treatment as soon as possible [47].

### Participants’ perspectives on knowledge about other non-infectious diseases that may affect the mother with TB infection and the unborn baby

Reducing maternal deaths is the key objective of the 2030 Sustainable Development Goals [5, 47]. The non-infectious diseases coupled with TB infection have implications for maternal health and mortality [5, 48]. The findings of the current study demonstrated that the participants are aware that diseases such as hypertension and diabetes further increase the risks of maternal and perinatal deaths. The participants furthermore indicated that swollen legs, nausea and vomiting, mood and anxiety disorders, tiredness and excessive sweating are normal reactions to pregnancy therefore a pregnant woman should not worry much about the symptoms because all were associated with hormonal imbalance. Despite the awareness of the risks of gestational hypertension and diabetes in pregnancy, older participants aged 35–40 were adamant about associating the risks with maternal and neonatal deaths. The finding could suggest that older pregnant women in the study need more information about the complications that might affect the mother and the baby. Studies in the United States show that women older than 35 faces increased gestational hypertension, gestational diabetes, preterm labour, and perinatal death [5]. Although the current study did not focus on knowledge about mental health and pregnancy, the literature indicates there are significant gaps and a lack of public awareness, research and access to screening, diagnosis, and treatment support among pregnant women experiencing mental distress [49]. This study could assume that a pregnant woman with mental imbalance might ignore symptoms of TB and early presentation to ANC service.

### Strengths and limitations of the study

The study was conducted in three districts that declared a high rate of TB disease in Limpopo province. In addition, a larger sample of 63 participants was selected to participate in individual interviews. This could suggest a wider generalization of the findings to all districts in Limpopo. However, due to geographic and socio-economic differences, the findings could not be generalized to the whole country, South Africa.

## Conclusion

The current study showed that pregnant women demonstrated general knowledge about TB and HIV infections, however, there is a huge gap in knowledge transfer and understanding about TB complications that might arise and for them to understand the adverse effect of the disease on the mother and the baby. The current study found that mothers lacked knowledge that TB during pregnancy could confer a high risk for maternal and infant morbidity. Pregnant women with TB disease need to be educated on the negative effects of non-adherence to TB treatment during pregnancy and neonatal periods. There is a need to educate pregnant women about the variant signs and symptoms of TB, HIV and COVID-19 infections since there is a misconception that the three diseases are similar. They should understand that the culture and beliefs that seem to be detrimental to the mother and the unborn baby could be modified to ensure the quality of life during pregnancy and to deliver a healthy baby. Health education and psychological support lessons are helpful in raising women’s awareness of MDR-TB prevention knowledge and improving their self-confidence toward delivering a baby who is not TB-infected. TB screening and assessment should be done concurrently with HIV screen at the first presentation to ANC service to avoid a unilateral focus on HIV infection, and progression to active TB disease if a woman has an infection. High-risk pregnant women showing signs and symptoms of TB should be thoroughly screened to ensure proper diagnosis so that treatment is commenced immediately. All pregnant women presenting for ANC service need to be educated that the benefits of TB/HIV treatment outweigh the potential risks of the medications.

## Data Availability

The datasets analysed during the current study are available through the study coordinator, Seani Adrinah Mulondo (Seani.mulondo@univen.ac.za) at the University of Venda on reasonable request.

## List of Abbreviations

AIDS: Acquired Immuno Disease Syndrome
ANC: Ante Natal Care
BANC: Basic Ante Natal Care
HIV: Human Immune Virus
LDoH: Limpopo Department of Health
MDR-TB: Multiple Drug Resistance-Tuberculosis
RPC: Research Publications Committee
SA: South Africa
SADC: South African Development Community
UNIVEN: University of Venda
WHO: World Health Organisation
